# Pathway of Toll-Like Receptor 7/B Cell Activating Factor/B Cell Activating Factor Receptor Plays a Role in Immune Thrombocytopenia *In Vivo*

**DOI:** 10.1371/journal.pone.0022708

**Published:** 2011-07-27

**Authors:** Qing Yang, Shuqian Xu, Xiaofang Li, Bo Wang, Xuping Wang, Daoxin Ma, Lei Yang, Jun Peng, Ming Hou

**Affiliations:** 1 Department of Parasitology, Shandong University School of Medicine, Jinan, China; 2 Haematology Oncology Centre, Qilu Hospital, Shandong University, Jinan, China; 3 Key Laboratory of Cardiovascular Remodeling and Function Research, Chinese Ministry of Education and Chinese Ministry of Public Health, Qilu Hospital, Shandong University, Jinan, China; 4 Hematology Department, Nanfang Hospital, Southern Medical University, Guangzhou, China; 5 Department of Traditional Chinese Medicine, Qilu Hospital, Shandong University, Jinan, China; University of Cape Town, South Africa

## Abstract

Immune thrombocytopenia (ITP) is an autoimmune disorder characterized by anti-platelet autoantibody-mediated platelet destruction. Antigen-presenting cell (APC) dysfunction is considered to play crucial roles in ITP. However, how APC affects autoreactive B cells in ITP is still unknown. Using a mouse model of immune thrombocytopenia, we demonstrated an increase in levels of TLR7 in splenic mononuclear cells (SMCs). Using both TLR7 agonist and TLR7 silencing lentivirus, we found stimulation of TLR7 decreased platelet counts and increased levels of platelet-associated IgG (PAIgG) in ITP mice, which correlates TLR7 with platelet destruction by autoantibodies. Levels of serum BAFF increased significantly in ITP mice and stimulation of TLR7 promoted secretion of BAFF. Among the three BAFF receptors, only BAFF receptor (BAFF-R) increased in ITP mice. However, activation of TLR7 showed no effect on the expression of BAFF receptors. These findings indicate that upregulation of TLR7 may augment BAFF secretion by APC and through ligation of BAFF-R promote autoreactive B cell survival and thus anti-platelet autoantibody production. The pathway of TLR7/BAFF/BAFF-R provides us with an explanation of how activation of APC affects autoantibody production by B cells in ITP and thus might provide a reasonable therapeutic strategy for ITP.

## Introduction

Immune thrombocytopenia (ITP) is an autoimmune disease manifested by immune-mediated platelet destruction and suppression of platelet production. Although several abnormalities involving the cellular mechanisms of immune modulation have been identified, development of autoantibodies against platelet glycoproteins remains central in the pathogenesis of ITP [1]. Increasing evidence suggests an important role of deviant APC in the pathophysiology of autoimmune diseases [2]. Targeting APC shows promising therapeutic effects in an animal model of rheumatoid arthritis (RA) [3]. In ITP patients, changes in number and function of APC have also been indicated [4]. Activation of APC is found to play a critical role in the pathogenic anti-platelet autoantibody response [4], [5]. However, how activation of APC affects autoantibody producing B cells is not well elucidated.

Toll-like receptors (TLRs) are type I transmembrane pattern-recognition receptors (PPRs) that have long been known to recognize highly conserved, pathogen-associated molecular patterns (PAMPs) [6]. TLRs are expressed on many cell types, especially APC [7]. They are key mediators of innate immunity and also regulate activation of adaptive immune system. Evidence suggests a role for TLRs in immune and inflammatory diseases and increasingly in autoimmunity [8]. Intracellularly localized TLR7 participates in APC activation and autoantibody production showing obvious importance in autoimmune diseases [9]. In 2006, using DNA microarrays, Sood et al. [10] found elevated levels of TLR7 in ITP patients. Increased levels of TLR7 in ITP were also indicated in our previous study using microarray analysis (data not published). Nevertheless, the role of TLR7 upregulation in APC in the pathophysiology of ITP is still unclear.

B cell activating factor (BAFF) is a member of the TNF superfamily and plays a major role in B cell survival [11]. BAFF has emerged as a crucial factor that modulates B cell tolerance and homeostasis. Elevated serum BAFF levels are involved in the pathogenesis of B cell-mediated autoimmune diseases such as systemic lupus erythematosus (SLE) [12], multiple sclerosis (MS) [13], systemic sclerosis (SS) [14] and RA [15]. BAFF binds to three receptors expressed on B cells: B cell maturation antigen (BCMA), transmembrane activator and calcium-modulating cyclophilin ligand interactor (TACI) and BAFF receptor (BAFF-R). Several lines of evidence indicate interaction between TLRs and BAFF or its receptors but few regarding the role of TLR7 [16], [17].

In the present study, we have explored the hypothesis that pathway of TLR7/BAFF/BAFF receptors accounts for APC affecting autoreactive B cells. The expression of TLR7, BAFF and BAFF receptors was detected in ITP using a thrombocytopenic mouse model. Then effects of TLR7 on platelet counts and levels of BAFF and BAFF-R in ITP mice were evaluated *in vivo* using TLR7 agonist and TLR7 silencing lentivirus. Our results correlate TLR7 with disease activity and indicate a role of TLR7/BAFF/BAFF-R pathway in the pathogenesis of ITP.

## Results

### Elevated levels of TLR7 in ITP mice

**Figure 1:**
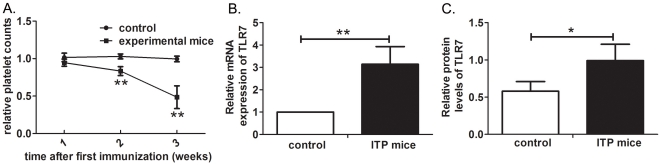
The platelet counts and levels of TLR7 in ITP mice and controls. (A) Changes in relative platelet counts of controls (n = 8) and experimental mice (n = 10). With rat platelet injection, experimental mice developed progressively thrombocytopenia. The relative platelet counts in experimental mice reached the minimum 3 weeks after the first immunization (0.49±0.15 vs 1, P = 0.000). There was no significant change in controls. SMCs were isolated from ITP mice (n = 10) and controls (n = 8) for RNA and protein extraction. (B) Results of real-time PCR analysis showed that the ratio of TLR7 mRNA in ITP mice compared to that of controls was 3.14 (P = 0.003). (C) Increased levels of TLR7 protein in ITP mice were revealed by western blot. The graph showed the densitometric quantitation of TLR7 to the housekeeping gene GAPDH. Bars represent SD, * represents P<0.05, ** represents P<0.01.

An ITP mouse model was developed according to Musaji [18]. The change of platelet counts was expressed as relative platelet count, i.e. the ratio of the platelet counts after immunization to the platelet counts before immunization. Fig. 1A showed that the decrease of relative platelet count was gradual and reached a maximum 3 weeks after the first rat platelet administration (0.49±0.15, P = 0.000). The experimental mice 3 weeks after the first immunization were referred to as ‘ITP mice’ in our later study.

To investigate whether TLR7 is activated in APC, SMCs were isolated from ITP mice and controls to determine the levels of TLR7. The relative mRNA expression of TLR7 was increased 3.14-fold (P = 0.003) in ITP mice compared to controls (Fig. 1B). The protein levels of TLR7 were also significantly increased in ITP mice compared with controls revealed by western blot (P = 0.033, Fig. 1C). Our data indicate that increased levels of TLR7 are involved in ITP mice.

### Effects of TLR7 on platelet counts

In order to explore the effects of TLR7 on ITP mice, TLR7 agonist (imiquimod) and TLR7 silencing lentivirus were used. The silencing efficiency of lentivirus was validated by transducing RAW264.7 cells *in vitro*. In our hands, more than 90% of RAW264.7 cells were transduced 3 days after transduction indicated by expression of GFP (Fig. S1A). Then RAW264.7 cells were harvested to determine the levels of TLR7 by real-time PCR and western blot. The relative TLR7 mRNA levels in RAW264.7 cells transduced with TLR7 silencing lentivirus was significantly decreased, only 3.82±0.62% relative to controls (P = 0.000) (Fig. S1B). Results of western blot showed that TLR7 silencing lentivirus can efficiently knockdown protein levels of TLR7 in RAW264.7 cells (P = 0.048, Fig. S1C), thus indicating that TLR7 silencing lentivirus can efficiently knockdown expression of TLR7 *in vitro*.

**Figure 2:**
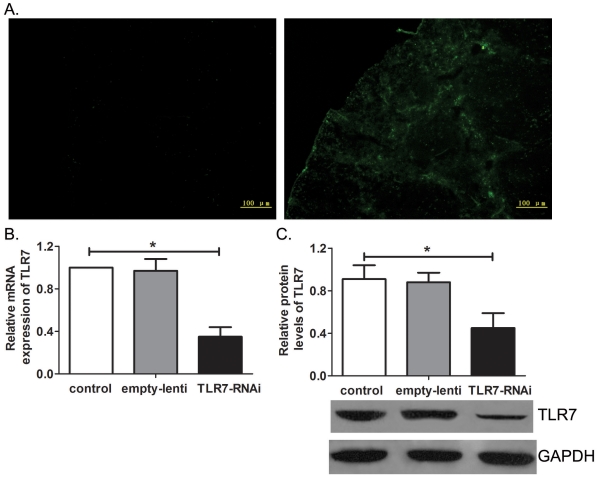
Analysis of TLR7 silencing lentivirus *in vivo*. The transduction efficiency of lentivirus *in vivo* was validated by GFP expression in spleen frozen sections from normal CBA mice (left) and ITP mice transduced with lentivirus (right), respectively. Scale bar, 100 µm. SMCs were isolated from ITP mice of group I (regarded as ITP mice, n = 10) group II (regarded as empty-lenti, n = 8) and group III (regarded as TLR7-RNAi mice, n = 8). Then SMCs were used to analyze the mRNA levels and protein levels of TLR7 by real-time PCR (B) and western blot (C), respectively. Bars represent SD, * represents P<0.05.

TLR7 silencing lentivirus was then used *in vivo*. The transduction efficiency of lentivirus *in vivo* was validated by GFP expression in spleen frozen sections (Fig. 2A). Four weeks after the first immunization, SMCs were isolated to determine the mRNA and protein levels of TLR7. Results of real-time PCR and western blot showed that TLR7 silencing lentivirus efficiently decreased the levels of TLR7 in ITP mice both in mRNA and protein levels (Fig. 2B and C), proving that TLR7 silencing lentivirus works efficiently *in vivo*.

**Figure 3:**
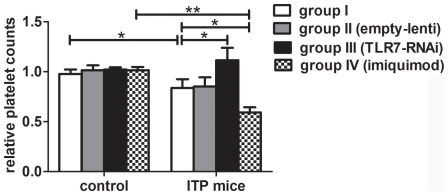
Effects of TLR7 on platelet counts. Empty lentiviral vector, TLR7 silencing lentivirus and imiquimod were used in both ITP mice and controls. The relative platelet counts of ITP mice were significantly increased in group III (n = 8) whereas decreased in group IV (n = 8) compared with group I (n = 10). No obvious changes were observed in the four groups of controls. Bars represent SD, * represents P<0.05, ** represents P<0.01.

The effects of TLR7 on platelet counts were then determined. TLR7 silencing lentivirus injection significantly increased the relative platelet counts (1.11±0.12 vs 0.84±0.09, P = 0.025) whereas imiquimod injection significantly decreased the relative platelet counts in ITP mice (0.59±0.05 vs 0.84±0.09, P = 0.034). However, no obvious changes were observed in different groups of controls (Fig. 3). These findings indicate that activation of TLR7 may negatively regulate platelet counts of ITP mice and thus is correlated to disease activity.

### Effects of TLR7 on PAIgG levels

**Figure 4:**
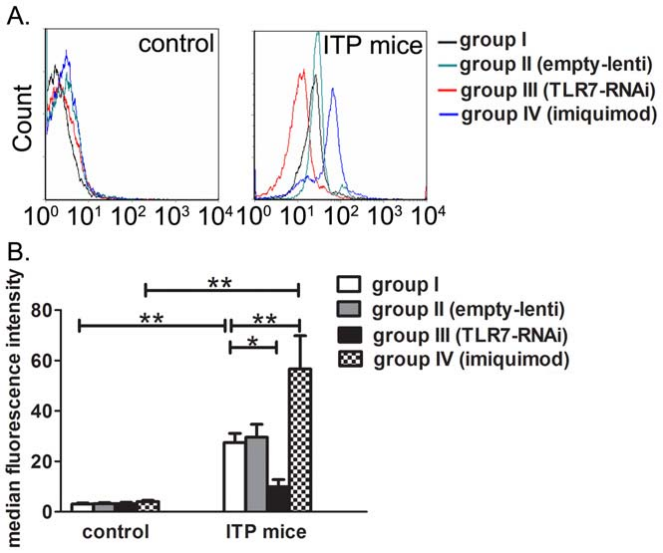
Effects of TLR7 on PAIgG levels. Platelets were collected from group I, group II (empty lentiviral vector injection), group III (TLR7 silencing lentivirus injection) and group IV (imiquimod injection) of ITP mice and controls. (A) Flow cytometry was performed to detect PAIgG levels on the surface of mice platelets of controls (left) and ITP mice (right). The MFI of PAIgG for different groups was demonstrated as histogram in (B). PAIgG levels were significantly increased in ITP mice compared with controls. Stimulation of TLR7 significantly increased PAIgG levels in ITP mice whereas inhibition of TLR7 significantly decreased PAIgG levels in ITP mice. No obvious changes were observed in the four groups of controls. Bars represent SD, * represents P<0.05, ** represents P<0.01.

As development of autoantibodies against platelets remains central in the pathogenesis of ITP, it is important to detect the effects of TLR7 on anti-platelet antibodies. Flow cytometry was performed in our study to detect levels of PAIgG and the median fluorescence intensity (MFI) was used to evaluate PAIgG levels. Four weeks after the first immunization, PAIgG levels were significantly increased in ITP mice compared to controls (27.44±3.68 vs 3.00±0.43, P = 0.002). Imiquimod injection significantly increased PAIgG levels (56.63±13.20, P<0.001) whereas TLR7 silencing lentivirus significantly decreased PAIgG levels (9.99±2.78, P = 0.026) in ITP mice. No obvious difference was observed in PAIgG levels among the four groups of controls (Fig. 4). Together, our results indicated that PAIgG levels were significantly increased in ITP mice and were promoted by TLR7.

### Effects of TLR7 on BAFF *in vitro* and *in vivo*

**Figure 5:**
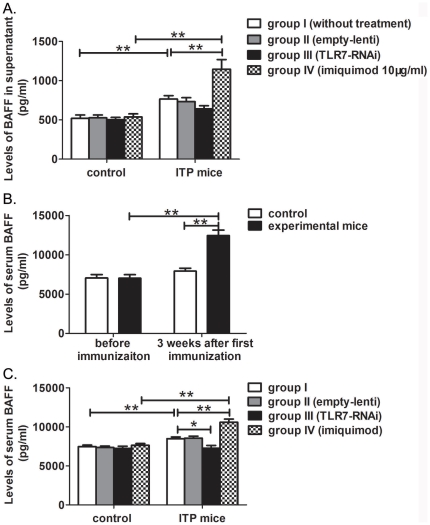
Effects of TLR7 on BAFF levels *in vitro* and *in vivo*. (A) Effects of TLR7 on BAFF levels *in vitro* were evaluated by SMCs culture. SMCs obtained from controls and ITP mice were divided into four groups and treated with or without empty lentiviral vector, TLR7 silencing lentivirus or imiquimod, respectively. The supernatant was collected and the levels of BAFF were determined by ELISA. (B) Serum samples were obtained from the experimental mice (n = 10) and controls (n = 8) before immunization and 3 weeks after the first immunization. A significant increase of serum BAFF was detected in ITP mice (At the time of 3 weeks after the first immunization, the experimental mice were regarded as ‘ITP mice’.) The difference between ITP mice and controls was also significant. (C) Effects of TLR7 on BAFF *in vivo* were determined using mice serum. Both controls and ITP mice were divided into four groups and treated with or without empty lentiviral vector, TLR7 silencing lentivirus or imiquimod, respectively. The serum from each group was obtained and the levels of BAFF were determined by ELISA. Bars represent SD, * represents P<0.05, ** represents P<0.01.

To elucidate how stimulation of TLR7 in APC affects anti-platelet antibody producing B cells, we detected the effects of TLR7 on BAFF levels both *in vitro* and *in vivo*. SMCs isolated from ITP mice and controls were cultured *in vitro* and the levels of BAFF in supernatant were detected by ELISA. Compared with controls, levels of BAFF in supernatant of SMCs were significantly increased in ITP mice (ITP mice: 766.09±43.03 pg/ml, controls: 519.19±43.95 pg/ml, P = 0.004). Imiquimod significantly increased the levels of BAFF in SMCs supernatant in ITP mice (1145.17±123.70 pg/ml vs 766.09±43.03 pg/ml, P<0.001) but not in controls. TLR7 silencing lentivirus did not cause a significant change in the levels of BAFF in either ITP mice or controls (Fig. 5A).

Effects of TLR7 on levels of BAFF were determined *in vivo*. Before immunization, the levels of serum BAFF in experimental mice and controls were 7015.27±454.33 pg/ml and 7043.45±435.89 pg/ml, respectively. There was no significant difference between the two groups (P = 1). Three weeks after the first immunization, the levels of serum BAFF in ITP mice were significantly elevated (12448.34±695.23 pg/ml vs 7015.27±454.33 pg/ml, P = 0.000) compared to the levels before immunization. A significant difference was also found between ITP mice (12448.34±695.23 pg/ml) and controls (7920.0±375.15 pg/ml) (P = 0.000). No significant difference was found in controls before and 3 weeks after saline injection (P = 0.224) (Fig. 5B).

Four weeks after the first immunization, the levels of serum BAFF in the four groups of ITP mice and controls were detected. At this time, the levels of serum BAFF in ITP mice were significantly increased compared to controls (ITP mice: 8481.66±211.54 pg/ml, control mice: 7463.67±198.55 pg/ml, P = 0.006). Imiquimod significantly increased the levels of serum BAFF in ITP mice (10578.43±417.54 pg/ml, P = 0.001) whereas TLR7 silencing lentivirus decreased the levels of serum BAFF in ITP mice (7258.36±359.17 pg/ml, P = 0.011). No obvious difference was observed in the four groups of controls (Fig. 5C). Our results indicate that levels of serum BAFF is elevated in ITP mice and stimulation of TLR7 promotes secretion of BAFF.

### Effects of TLR7 on BAFF receptors

**Figure 6:**
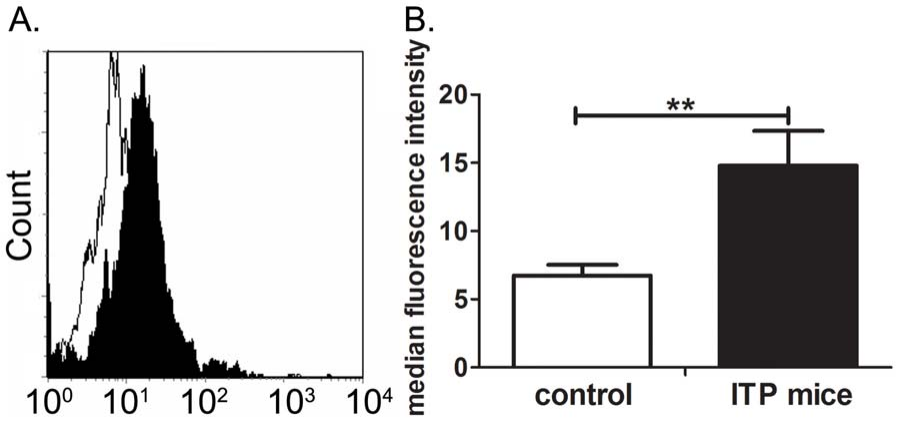
Flow cytometry analysis for the expression of BAFF-R in ITP mice and controls. Peripheral blood samples were obtained from ITP mice (n = 10) and controls (n = 8). Two-color staining was carried out on blood samples. (A) A representative experiment shown here indicated a significant increase of BAFF-R in ITP mice (black) compared with controls (blank). (B) The MFI of BAFF-R is presented as mean ± SD in histogram. Bars represent SD, ** represents P<0.01.

To investigate through which BAFF receptor elevated serum BAFF affects B cells, the levels of the three BAFF receptors on B cells in peripheral blood of ITP mice and controls were determined. Results of flow cytometry showed that among the three receptors, only BAFF-R increased significantly in ITP mice when compared to controls (P = 0.006, Fig. 6). No significant difference in BCMA and TACI levels were detected. The median fluorescence intensity (MFI) of BCMA and TACI in ITP mice was 8.45±1.24 and 9.55±0.97, respectively. The MFI of BCMA and TACI in controls was 8.07±0.99 and 9.14±1.12, respectively (data not shown). Therefore, elevated BAFF promoting B cell survival through BAFF-R and suggests that the pathway of TLR7/BAFF/BAFF-R plays a role in ITP.

**Figure 7:**
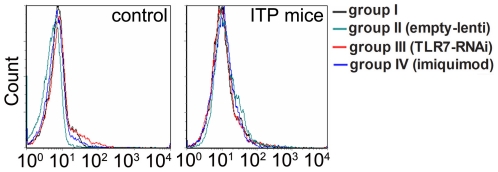
Effects of TLR7 on BAFF-R levels. Both controls and ITP mice were divided into four groups and treated with or without empty lentiviral vector, TLR7 silencing lentivirus or imiquimod, respectively. Blood samples were collected from each group and the levels of BAFF-R on B cells were determined by flow cytometry. Stimulation or inhibition of TLR7 made no significant difference in the levels of BAFF-R in both control mice (left) and ITP mice (right).

As previous studies showed that TLR7 might influence the expression of BAFF receptors directly [17], effects of TLR7 on BAFF-R were then determined. Results of flow cytometry showed no significant difference after stimulation or inhibition of TLR7 in either controls or ITP mice (Fig. 7). These findings showed that no direct interaction was found between TLR7 and BAFF-R in ITP mice. Altogether, our results indicate that stimulation of TLR7 in APC enhances secretion of BAFF by APC and thus through ligation of BAFF-R promotes survival of autoreactive B cells and contributes to autoantibody-mediated platelet destruction in ITP mice.

## Discussion

In this study, a thrombocytopenic mouse model described by Musaji [18] was used to explore the mechanism of APC affecting autoreactive B cells. Elevated levels of PAIgG detected in ITP mice indicate that the drop in platelet counts was due to the anti-platelet autoantibody-mediated autoimmune response [18]. Additionally, presence of autoantibodies makes this thrombocytopenic mouse model suitable for our study on mechanism between APC activation and B cell autoantibody production. To construct this thrombocytopenia model, rat platelets were injected into CAB mice. Rat platelets injection may promote an anti-platelet response and also additional immune responses. Saline injection in control mice may not be a good control. However, results of Musaji and ours showed that anti-platelet autoantibody production [18] and increased TLR7 and BAFF levels (data supplied on request) are specific to CBA mice but not in BALB/c mice. This indicates that the changes observed in our study are mainly due to the anti-platelet response.

TLR7 was found to be significantly increased in ITP mice. Elevated levels of TLR7 have also been detected in autoimmune diseases mediated by autoreactive B-cell clones such as SLE [19] and even in ITP [10]. Previous studies show that, in SLE, TLR7 is necessary and sufficient for antibody production. Inhibition of TLR7 reduces anti-RNA autoantibody production, glomerular IgG levels and glomerular macrophage infiltration and thus attenuates glomerulonephritis in lupus mice and represents a potential therapeutic target in lupus [9], [20]. Effects of TLR7 on the pathology of ITP were determined in our study. In ITP mice, activation of TLR7 negatively-regulates platelet counts and increases PAIgG levels. Our results indicate that TLR7 is correlated with disease activity in ITP and contributes to autoantibody-mediated platelet destruction. But the potential source of TLR7 ligand in ITP is still unknown. A pathogen as a source of ssRNA or human mRNA in an immune complex may participate in TLR7 stimulation. Additionally, how stimulation of TLR7 in APC affects production of anti-platelet antibodies by B cells is not well elucidated in ITP.

Previous studies show that activation of TLRs might influence expression or secretion of BAFF. Stimulation of TLR4 augmented BAFF expression in spleen cells *in vitro* by inducing reactive oxygen species and increased the total number of B cells in the spleen *in vivo* [21]. TLR3 was found to significantly up-regulate BAFF expression in human bronchial epithelial cells via an autocrine pathway involving IFN-β. Thus through local accumulation, activation and Ig synthesis by B cells in the airway, TLR3 plays an important role in airway immune responses [16]. However, contrary results were reported elsewhere. Elevated levels of BAFF were detected in RA patients. But none of the ligands of TLR2, TLR4 and TLR9 induced the de novo synthesis and release of BAFF by synoviocytes. In addition, IFN-γ-induced BAFF synthesis was inhibited by stimulation of TLR2, 4, 9 [22]. In our study, significantly elevated levels of serum BAFF were detected in ITP mice. This result suggests that BAFF participates in the pathogenesis of ITP. This is in accordance with the results of previous studies concerning other autoimmune disorders [12]–[14]. In conclusion, our results showed that levels of serum BAFF was positively regulated by TLR7 in ITP mice indicating that elevated TLR7 augmented secretion of BAFF by APC in ITP.

BAFF, a crucial homeostatic cytokine for B cells, exerts its role through binding to its receptors: BCMA, TACI and BAFF-R. A proliferation inducing ligand (APRIL), a cytokine homologous to BAFF, can bind to BCMA and TACI but not BAFF-R which makes BAFF-R a specific receptor for BAFF [23]. BAFF-R, expressed on a wide range of B cell subsets, is the predominant receptor that mediates BAFF-dependent B cell signaling and plays critical roles in controlling peripheral B cell survival [3]. BAFF-R was often found increased in autoantibody related diseases [24]. However, some studies showed that expression of BAFF-R did not change or even decrease in autoimmune diseases including SLE, SS, MS and RA [15], [25]–[27]. Results of Zhou et al. showed that mRNA expression of BAFF-R did not change in peripheral blood mononuclear cells but increased in SMCs in ITP patients [28]. Unfortunately, the protein levels of BAFF-R were not detected in their research. Here, our study indicated a significant increase of BAFF-R while no changes in BCMA and TACI on peripheral B cells in ITP mice. Our results suggested that excess BAFF promoted autoreactive B cell survival by binding to BAFF-R in ITP.

Except for the effects on expression of BAFF, stimulation of TLRs may also influence expression of BAFF receptors directly. TLRs, together with BAFF receptors were shown to regulate the function and homeostasis of B cells. Activation of TLR9 strongly elevated TACI levels on B cells in a MyD88-dependent pathway whereas TLR4 ligand might increase both TACI and BAFF-R levels in MyD88-dependent and independent pathways [17]. In lupus-like mice, activation of TLR7/9 led to a strong upregulation of TACI [29]. In our study, either activation of TLR7 or inhibition of TLR7 made no significant difference in the expression of BAFF-R. This indicates that TLR7 does not interact with BAFF-R directly in ITP but exerts its role through interaction with BAFF.

Additionally, B cells also express TLR7. Stimulation of TLR7 may augment BAFF secretion by B cells and thus through ligation of BAFF receptors promotes autoreactive B cell survival. To separate this direct effect of B cells from the indirect effect via APC described in our work, specific stimulation or silencing of TLR7 in APC is needed.

In summary, levels of TLR7 are elevated in ITP mice. Stimulation of TLR7 is related to disease activity by promoting platelet destruction through anti-platelet antibodies in ITP mice. The levels of serum BAFF are increased in ITP mice and stimulation of TLR7 promotes secretion of BAFF. Among the three BAFF receptors, only BAFF-R is elevated in ITP mice. No direct interaction between TLR7 and BAFF-R is detected. These results indicate a role of TLR7/BAFF/BAFF-R in the pathogenesis of ITP. The pathway of TLR7/BAFF/BAFF-R provides us with an appropriate explanation of how activation of APC affects autoreactive B cells and autoantibody production in ITP and thus regulating this pathway might provide a reasonable therapeutic strategy for ITP.

## Materials and Methods

### Lentiviral silencing vector

Lentiviral silencing vector targeting mouse TLR7 (NM_133211) was constructed and packaged by Genechem. The target sequence of the TLR7 silencing vector was 5′-GATCTGCCATCCAGCTTACAT-3′ [30]. RAW264.7 cells [31] were transduced with lentivirus at a multiplicity of infection (MOI) of 20 in serum free medium containing 10 µg/ml polybrene in 6-well plates. After 8 h, DMEM medium containing 10% FBS was added to the cells. Three days later, the transduction efficiency was evaluated by expression of GFP (Both of the lentiviral vectors contain a GFP expression cassette) and the RAW264.7 cells were collected for RNA and protein extraction.

### Mouse model

Female CBA mice about 7 weeks old were purchased from Shanghai Slac Laboratory Animal Limited Company and the study was approved by the Ethics Committee of Shandong University School of Medicine (No. 005 in 2008 for Animal Ethics Approval). This model was first described by Musaji [18]. Briefly, blood of Wistar rats was collected from jugular vein. Platelet-rich supernatant was prepared by successive centrifugations. Platelets were pelleted from this supernatant and washed as appropriate. Then rat platelets were used to immunize experimental mice. Rat platelets 10^8^ in 0.5 ml saline were first administered intraperitoneally, followed by 0.5×10^8^ platelets weekly. Controls were injected with saline alone. Blood was collected from the retro-orbital plexus of ether-anesthetized CBA mice and platelets were counted by Hemavet 950 Animal Haematology Analyzer (Drew Scientific).

Three weeks after the first immunization, treated mice with minimum platelet counts were regarded as ‘ITP mice’. The ITP mice and controls were divided into four groups, respectively: group I received rat platelets (n = 10) or saline (n = 8) injection as before, group II received injection of rat platelets (n = 8) or saline (n = 8) once plus tail intravenous injection of 10^7^ transduction unit (TU) empty lentiviral vector, group III received rat platelets (n = 8) or saline (n = 8) injection once plus tail intravenous injection of 10^7^ transduction unit (TU) lentiviral silencing vector of TLR7 and group IV received rat platelets (n = 8) or saline (n = 8) injection once plus 25 µg imiquimod (TLR7 agonist) (InvivoGen) every other day [32]. Effects of TLR7 were detected one week later (i.e. four weeks after the first immunization).

### Splenic mononuclear cells (SMCs)

SMCs were isolated from spleens of mice using 1.092 g/ml Ficoll-Hypaque (TBD) gradient centrifugation (2 000 rpm for 20 min, 20°C). The isolated SMCs were washed twice with PBS for culture, RNA and protein extraction. For culture, the isolated SMCs were diluted to 10^6^/ml and seeded into RPMI 1640 medium supplemented with 10% FBS and 10 ng/ml IL-2. The SMCs in culture were divided into four groups: group I (without treatment), group II (transduced with empty lentiviral vector), group III (transduced with TLR7 silencing lentivirus) and group IV (imiquimod 10 µg/ml). Then on day 3 the supernatant of SMCs was collected for ELISA analysis.

### Quantitative real-time PCR analysis

Total RNA was isolated from cells with TRIzol reagent (Invitrogen). The amount of RNA was determined using ultraviolet spectrophotometer DU800 (Beckman) and normalized to 1 µg/ml. Total RNA was converted into cDNA using the PrimeScript™ RT reagent kit (Takara). Real-time PCR was performed for TLR7 and GAPDH on Lightcycler 2.0 (Roche) using SYBR Premix Ex Taq (Takara). The sequences of the amplification primers for TLR7 and GAPDH are as follows: TLR7 forward, 5′-CCTAGGACATGCCTTGGTACCTG-3′, TLR7 reverse, 5′-CCCACCAATCTGAGCCATGA-3′; GAPDH forward, 5′-TGTGTCCGTCGTGGATCTGA-3′, GAPDH reverse, 5′-TTGCTGTTGAAGTCGCAGGAG-3′. LightCycler Software 4.0 (Roche) was used to determine Ct value. All PCR products were visualized by 2% agarose gel electrophoresis stained by ethidium bromide. The mRNA expression of TLR7 was normalized to GAPDH relative to control using 2^−ΔΔCt^ method [33].

### Western blot

Cells were lysed in RIPA Lysing Buffer (Beyotime). The total protein was extracted after homogenization and centrifugation. The protein concentration was determined using BCA Protein Assay Kit (Pierce). GAPDH was used to normalize the amount of protein in all western blot experiments. Proteins were loaded onto SDS-PAGE and transferred to a nitrocellulose membrane. The membrane was probed with antibodies to TLR7 (Abcam) and GAPDH (Jingmei). The secondary antibody used was HRP anti-rabbit antibody (Jingmei). Protein bands were visualized with ECL system (Pierce).

### ELISA

Peripheral blood samples from CBA mice clotted for 2 h at room temperature and were centrifuged to obtain serum. Supernatant from SMCs was collected by centrifugation. Then levels of BAFF in serum and supernatant were measured by Quantikine Mouse BAFF/BLyS/TNFSF13B Immunoassay (R&D) in 96-well microtiter according to manufacture's instruction. The lower detection limit of the assay was 4.3 pg/ml.

### Flow cytometry

For determination of BAFF receptors, peripheral blood samples of CBA mice 100 µl were incubated with 2.5 µl PerCP-Cy5.5 anti-mouse B220 and 0.625 µl PE anti-mouse TACI or 5 µl Alexa Flour 647 anti-mouse BAFF-R (eBioscience) or 20 µl monoclonal anti-mouse BCMA-Fluorescein (R&D) for 30 min. B220 was used to label B cells and was co-stained with the three BAFF receptors, respectively. Then 2 ml 1×FCM Lysing Solution (MultiSciences Biotech) was added and the samples were incubated in the dark for 10 min. For detection of platelet-associated IgG (PAIgG), 2×10^7^ mice platelets were incubated with 1 µl FITC anti-mouse IgG (H+L) for 30 min.

After centrifuge to remove supernatant, the cell and platelet pellets were washed with PBS twice. Finally, 300 µl PBS were added to resuspend the cell and platelet pellets and the samples were determined by flow cytometer (BD Biosciences). The data were analyzed using FCS Express V3.

### Statistical analysis

Data were expressed as mean ± SD. Statistical significance was determined by one-way analysis of variance (ANOVA) using the Statistical Package for the Social Sciences (SPSS) version 13.0. P value less than 0.05 was considered statistically significant.

## Supporting Information

Figure S1 Knockdown of TLR7 by silencing lentivirus in vitro. Lentivirus expressing shRNA targeting mouse TLR7 and empty lentiviral vector were transduced into RAW264.7 cells and regarded as TLR7-RNAi and empty-lenti, respectively. (A) Three days after transduction, more than 90% of RAW264.7 cells were transduced as indicated by expression of GFP. Scale bar, 200 µm. Then RAW264.7 cells were harvested for RNA and protein extraction. (B) The mRNA levels of TLR7 were detected by real-time PCR. The data were presented as percentage in gene expression normalized to GAPDH relative to controls. (C) The protein levels of TLR7 were determined by western blot. The graph showed the densitometric quantitation of TLR7 to the housekeeping gene GAPDH. Expression of TLR7 in both mRNA and protein levels was efficiently knocked down by TLR7 silencing lentivirus in RAW264.7 cells. Bars represent SD, * represents P<0.05, ** represents P<0.01.
(TIF)
