# Supplementary figures and images for: Pathway of Toll-Like Receptor 7/B Cell Activating Factor/B Cell Activating Factor Receptor Plays a Role in Immune Thrombocytopenia In Vivo
Source: PLoS One. 2011 Jul 27;6(7):e22708. doi: 10.1371/journal.pone.0022708 (PMC3144916; doi:10.1371/journal.pone.0022708)

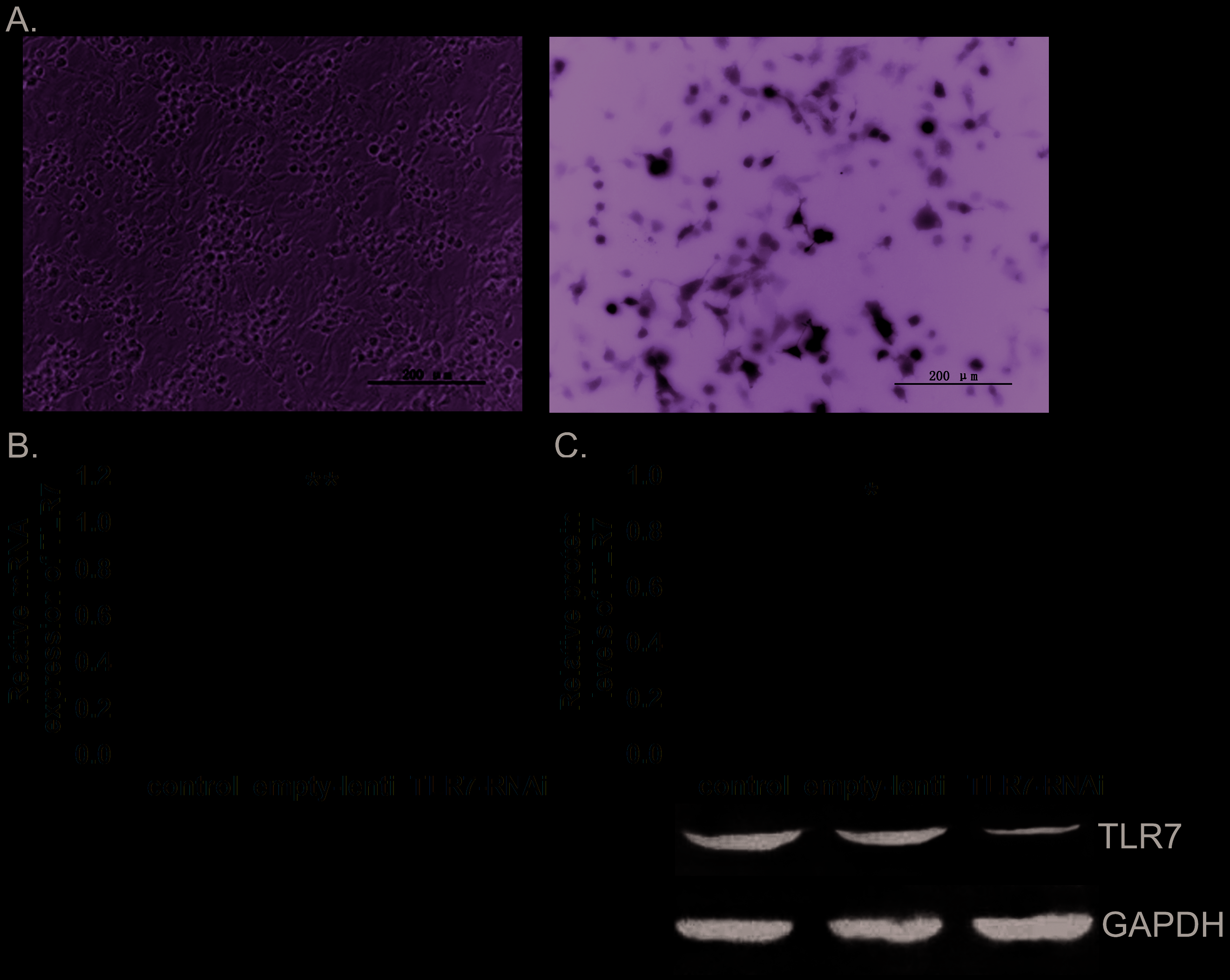

Supplement: Figure S1 — Knockdown of TLR7 by silencing lentivirus in vitro. Lentivirus expressing shRNA targeting mouse TLR7 and empty lentiviral vector were transduced into RAW264.7 cells and regarded as TLR7-RNAi and empty-lenti, respectively. (A) Three days after transduction, more than 90% of RAW264.7 cells were transduced as indicated by expression of GFP. Scale bar, 200 µm. Then RAW264.7 cells were harvested for RNA and protein extraction. (B) The mRNA levels of TLR7 were detected by real-time PCR. The data were presented as percentage in gene expression normalized to GAPDH relative to controls. (C) The protein levels of TLR7 were determined by western blot. The graph showed the densitometric quantitation of TLR7 to the housekeeping gene GAPDH. Expression of TLR7 in both mRNA and protein levels was efficiently knocked down by TLR7 silencing lentivirus in RAW264.7 cells. Bars represent SD, * represents P<0.05, ** represents P<0.01. (TIF) [file pone.0022708.s001.tif]
